# Evaluation of the Adequacy of Eating Disorder Training in Psychiatric Residency Programs in Turkey: A Cross-Sectional Study

**DOI:** 10.1192/j.eurpsy.2025.915

**Published:** 2025-08-26

**Authors:** B. Akan, S. Kukurt, B. Ozel, G. Aşut

**Affiliations:** 1 Psychiatry, Pamukkale University, Denizli; 2 Psychiatry, Bezmialem Vakif University, Istanbul; 3 Psychiatry, Baskent University, Ankara, Türkiye

## Abstract

**Introduction:**

Eating disorders (ED) are serious mental illnesses affecting both physical and psychological health. In Turkey, no systematic research has been conducted on the adequacy of ED education in psychiatric residency programs. This study aims to evaluate the perceptions of psychiatric residents regarding the adequacy of their ED training and to offer suggestions for curriculum development.

**Objectives:**

The primary objective is to assess psychiatric residents’ perceptions of the adequacy of their training in EDs and compare this with their training in other psychiatric disorders. Additionally, the study aims to propose improvements to the training curriculum.

**Methods:**

A cross-sectional online survey was conducted among 133 psychiatric residents across Turkey. Participants were recruited through social media groups for psychiatric residents, and data were collected in August 2024. The minimum sample size was determined to be 111, based on power analysis. The survey included sociodemographic questions (age, gender, institution type, years of residency) and questions regarding ED training, supervision experience, and other psychiatric disorders. Categorical variables were summarized using percentages and frequencies, while continuous variables were presented as means and medians. McNemar’s test was used to assess differences in training and self-efficacy between two diagnostic groups. Ethical approval was obtained from Başkent University (KA24/297).

**Results:**

The mean age of the participants was 28.3 years (SD: 2.1), and the average duration of their residency was 2.6 years (SD: 1.1). Regarding ED training, 46.2% (n=62) reported receiving theoretical lessons, while 84.2% (n=112) reported no supervision. Additionally, 56.4% (n=75) considered their ED training insufficient, and 55.2% (n=73) felt inadequately prepared to manage EDs, which was significantly different from other disorders (for psychotic disorders: χ² = 34.225, p <.001; χ² = 29.167, p <.001, anxiety disorders: χ² = 9.031, p =.003; χ² = 35.027, p <.001, sleep disorders: χ² = 18.581, p <.001; χ² = 34.028, p <.001, neurocognitive disorders: χ² = 34.028, p <.001; χ² = 30.031, p <.001).

**Conclusions:**

This study demonstrates that psychiatric residents in Turkey, perceive their training in EDs as inadequate. This may stem from the limited time allocated to ED education, lack of supervision, and insufficient clinical experience. The perception that EDs are less common and complex could contribute to limited resources being allocated to their education. These inadequacies may affect residents’ ability to manage ED patients effectively. A comprehensive review of training programs, including more practical experience and supervision, could address these deficiencies.

**Disclosure of Interest:**

None Declared

